# pH-Mediated Microbial and Metabolic Interactions in Fecal Enrichment Cultures

**DOI:** 10.1128/mSphere.00047-17

**Published:** 2017-05-03

**Authors:** Zehra Esra Ilhan, Andrew K. Marcus, Dae-Wook Kang, Bruce E. Rittmann, Rosa Krajmalnik-Brown

**Affiliations:** a Biodesign Swette Center for Environmental Biotechnology, Arizona State University, Tempe, Arizona, USA; b School of Life Sciences, Arizona State University, Tempe, Arizona, USA; c Biodesign Center for Fundamental and Applied Microbiomics, Arizona State University, Tempe, Arizona, USA; d School of Sustainable Engineering and the Built Environment, Tempe, Arizona, USA; University of Wisconsin—Madison

**Keywords:** alkalinity, bacterial diversity, lactate utilizers, microbial communities, microbial fermentation, propionate producers, substrate type

## Abstract

pH and fermentable substrates impose selective pressures on gut microbial communities and their metabolisms. We evaluated the relative contributions of pH, alkalinity, and substrate on microbial community structure, metabolism, and functional interactions using triplicate batch cultures started from fecal slurry and incubated with an initial pH of 6.0, 6.5, or 6.9 and 10 mM glucose, fructose, or cellobiose as the carbon substrate. We analyzed 16S rRNA gene sequences and fermentation products. Microbial diversity was driven by both pH and substrate type. Due to insufficient alkalinity, a drop in pH from 6.0 to ~4.5 clustered pH 6.0 cultures together and distant from pH 6.5 and 6.9 cultures, which experienced only small pH drops. Cellobiose yielded more acidity than alkalinity due to the amount of fermentable carbon, which moved cellobiose pH 6.5 cultures away from other pH 6.5 cultures. The impact of pH on microbial community structure was reflected by fermentative metabolism. Lactate accumulation occurred in pH 6.0 cultures, whereas propionate and acetate accumulations were observed in pH 6.5 and 6.9 cultures and independently from the type of substrate provided. Finally, pH had an impact on the interactions between lactate-producing and -consuming communities. Lactate-producing *Streptococcus* dominated pH 6.0 cultures, and acetate- and propionate-producing *Veillonella*, *Bacteroides*, and *Escherichia* dominated the cultures started at pH 6.5 and 6.9. Acid inhibition on lactate-consuming species led to lactate accumulation. Our results provide insights into pH-derived changes in fermenting microbiota and metabolisms in the human gut.

**IMPORTANCE** The human gut is a dynamic environment in which microorganisms consistently interact with the host via their metabolic products. Some of the most important microbial metabolic products are fermentation products such as short-chain fatty acids. Production of these fermentation products and the prevalence of fermenting microbiota depend on pH, alkalinity, and available dietary sugars, but details about their metabolic interactions are unknown. Here, we show that, for *in vitro* conditions, pH was the strongest driver of microbial community structure and function and microbial and metabolic interactions among pH-sensitive fermentative species. The balance between bicarbonate alkalinity and formation of fatty acids by fermentation determined the pH, which controlled microbial community structure. Our results underscore the influence of pH balance on microbial function in diverse microbial ecosystems such as the human gut.

## Abstract (precis)

The human gut is a dynamic environment in which microorganisms consistently interact with the host via their metabolic products. Some of the most important microbial metabolic products are fermentation products such as short-chain fatty acids. Production of these fermentation products and the prevalence of fermenting microbiota depend on pH, alkalinity, and available dietary sugars, but details about their metabolic interactions are unknown. Here, we show that, for *in vitro* conditions, pH was the strongest driver of microbial community structure and function and microbial and metabolic interactions among pH-sensitive fermentative species. The balance between bicarbonate alkalinity and formation of fatty acids by fermentation determined the pH, which controlled microbial community structure. Our results underscore the influence of pH balance on microbial function in diverse microbial ecosystems such as the human gut.

## INTRODUCTION

Gut microorganisms use a variety of fermentative pathways to harvest energy, and the pathways utilized depend on many factors, including pH and available fermentation substrates (1, 2). The pH varies from about 5 to 7 along the human colon (3), with the type and abundance of fermentation products, bicarbonate secretion by colonic epithelial cells, and absorption of microbial metabolites by host epithelial cells (4–6). The pHs of the ascending (pH 5.4 to 5.9) and transverse (pH ≈6.2) colons are lower than those of the descending and rectosigmoid colons (pH 6.6 to 6.9) (7). Many of the host’s dietary nutrients are substrates for microbial metabolism, such as oligosaccharides or simple sugars, and the nutrients have an impact on pH as they promote acid production via fermentation (8). Colonic pH along with gut microorganisms deviates from normal during gastrointestinal diseases, such as colorectal cancer (9), inflammatory bowel disease (4), and constipation (10). Exogenous factors, such as the use of proton pump inhibitors (11) or changes in the gut anatomy due to bariatric surgery (12), also alter gut pH. It is likely that an abnormal pH in the gut will alter microbiota composition and its metabolism (12, 13).

pH imposes selective pressure on microbial growth and metabolism. While some bacteria, such as *Bacteroides*, can grow over a wide range of pH values (14), others, such as *Veillonella* and *Streptococcus*, are inhibited by acidic pH (15), although some species from these genera can thrive (15). Furthermore, pH is an important determinant of the distribution of major fermentation end products. For example, butyrogenic reactions occur at mildly acidic pH (pH 5.5) (1), propionate-producing reactions often occur at neutral pHs (16), and acetogenic reactions occur at various pHs depending on the microbial species producing them (16). Shifts in microbiota composition and metabolism can affect colonic function; for instance, at mildly acidic pH (pH 5.5), butyrogenic *Faecalibacterium* and *Roseburia* grow better and produce more butyrate than they do at approximately neutral pH (pH 6.7) (1) and, hence, change colonic function by feeding colonocytes and protecting against hydrogen peroxide-induced DNA damage (17). On the other hand, propionate production can be slightly inhibited at mildly acidic pH (pH 5.5) as a result of limited growth of propionate-producing species, such as *Bacteroides* (1). Propionate, the second preferred energy source for colonocytes after butyrate (18), has anti-inflammatory properties and can play a key role in the treatment of inflammatory bowel diseases (19).

Dietary carbohydrates have a major effect on the structure and function of microbial communities in the human gut (2, 20). Prebiotic polysaccharides such as inulin and pectin can stimulate the growth of certain microbial species under *in vitro* conditions, and the community structures depend on starting pH (2). Monosaccharides and disaccharides can be found in the diet in their monomeric forms (21) and as building blocks of common polysaccharides such as cellulose, inulin, and oligofructose (21). In a typical diet, polysaccharides constitute 40% of the total calories (22), and an increase in the consumption of high-fructose corn syrup and prebiotics such as inulin and oligofructose increases the ratio of dietary fructose to glucose (23, 24). On average, an adult consumes 37 to 100 g of fructose per day (25). The major sources of cellobiose are dietary plant compounds; as a disaccharide, cellobiose is less abundant in the diet.

Among the components of dietary carbohydrates, glucose fermentation by enteric bacteria under different pH conditions has been well documented (26–29). In contrast to glucose, fermentation of other saccharides, such as fructose and cellobiose, by human gut microbiota has had minimal attention. Fructose is more accessible to the colonic microbiota than glucose because it is absorbed less efficiently than glucose in the small intestine (21). Cellobiose is a disaccharide, and its bioavailability in the colon is limited by its hydrolysis by the gut microbiota (30). The relative roles of these organic substrates in microbial community composition and their functions remain unclear and demand more investigation. Additionally, to date, the majority of *in vitro* studies (1, 2) that characterize microbial growth and function have neglected the impact of changes in pH and alkalinity on microbial and metabolic interactions in mixed microbial communities.

Here, we address the effects of pH and carbohydrates on the structure and function of anaerobic microbial communities derived from a fecal slurry obtained from a healthy human using an *in vitro* batch fermentation model. *In vitro* batch models offer great resolution to the investigation of microbe-microbe interactions, gut microbiome functionality, and insight into the microbial metabolism of complex substrates into fermentation products (31). We carried out batch *in vitro* experiments using glucose, fructose, or cellobiose as the organic substrate and initial pH values of 6.0, 6.5, or 6.9; these values range from normal to unusually low values associated with gastrointestinal abnormalities in the distal colon. We evaluated impacts on the microbial communities’ structure and metabolic end products. We applied electron-equivalent mass balances to quantify the effects of pH and substrate on community function.

## RESULTS AND DISCUSSION

### Mixed community structure depends more on pH than on carbon type.

**FIG 1:**
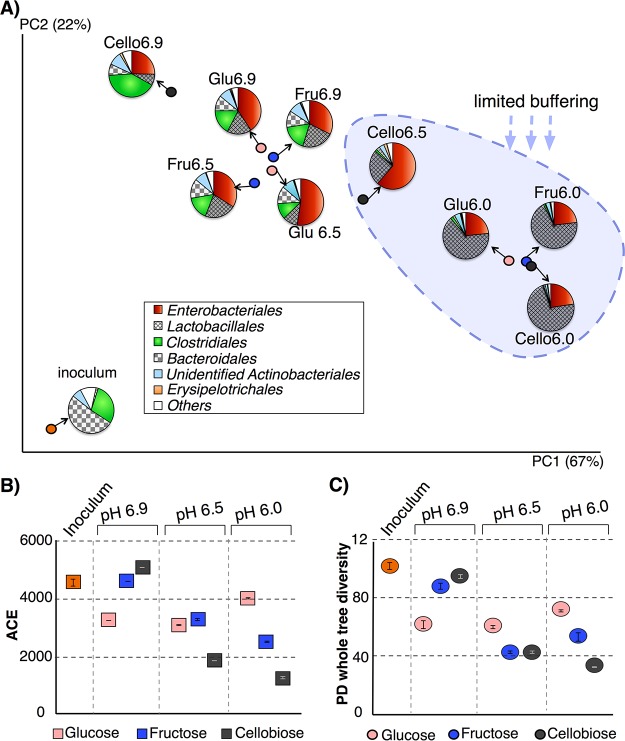
(A) Weighted UniFrac (32) analysis visualized on principal coordinates shows that mainly the initial pH along with buffering determined the main phylotypes that drove the community structures in the system. Each circle represents microbial communities from pooled DNA samples from triplicate reactors. (B and C) Abundance-based coverage estimator (ACE) (33) (B) and PD whole-tree (34) (C) indices calculated from 16S rRNA gene sequences for inoculum and pH 6.0, 6.5, and 6.9 cultures.

In order to identify whether pH or the substrate exerted greater selective pressure on the microbial communities, we compared weighted and unweighted UniFrac distances (32) of the glucose (Glu), fructose (Fru), and cellobiose (Cello) cultures at initial pHs of 6.0, 6.5, and 6.9 to each other and visualized the results of weighted UniFrac distances on principal coordinates, shown in Fig. 1A. Unweighted UniFrac analysis, which provides information on changes in taxa that are not abundant, did not yield clusters based on pH or carbon source. Weighted UniFrac analysis, which relies on the abundance of phylotypes in addition to their presence or absence, provides information regarding the most abundant taxa. Since we had enrichment cultures from the same inoculum, weighted UniFrac analysis reflected changes in operational taxonomic unit (OTU) abundances and was more suitable for our data set.

When we investigated whether pH or the organic substrate had a greater impact on clustering, as shown in Fig. 1A, we did not observe a statistical difference based on the organic substrate utilized. However, samples formed two distinct clusters based on pH on principal coordinate 1 (PC1), which explained 67% of the variation in the whole data set. The first cluster was composed of pH 6.0 cultures, and the second one was composed of pH 6.5 and 6.9 cultures. pH 6.0 cultures were statistically distant from pH 6.9 cultures (analysis of similarity [ANOSIM]; *P* = 0.0403, *R* = 0.82). We did not observe this significant difference between pH 6.5 and pH 6.0 cultures, possibly due to the small sample size. The pH 6.5 and 6.9 cultures overlapped on PC1, although the pH 6.9 cultures were higher on PC2, which explained 22% of the variation in the whole data set. pH 6.9 cultures, especially Cello6.9, appeared the closest to the inoculum on PC1. The pH of the inoculum was 7.1, and its data profile most closely matches those of the pH 6.9 cultures.

The medium’s buffering capacity played a substantial role in the development of microbial communities. As shown in Table 1, 30 mM HCO_3_^−^ provided 3.2 to 3.5 mM alkalinity to pH 6.0 cultures, whereas it provided 10.7 to 11.9 and 26.8 to 31.7 mM alkalinity to the pH 6.5 and 6.9 cultures, respectively. Greater bicarbonate buffering at pH 6.5 and 6.9 led to much smaller drops in pH in 72 h, and this correlated with the development of similar microbial communities. Since pH 6.9 cultures provided sufficient buffering and did not experience significant drops in pH (<0.4), the microbial communities remained more similar to the inoculum (neutral pH) than the others. The concentration of acids produced in pH 6.0 cultures and Cello6.5 cultures by 72 h far exceeded the concentration of alkalinity, as summarized in Table 1. Therefore, rapid fermentation led to insufficient buffering, and the pH dropped dramatically (*P* < 0.05). The substantial drop in the pH appeared to promote the survival of only acid-tolerant species, a trend that drove microbial communities away from pH 6.5 and 6.9 cultures.

**TABLE 1:** Amount of biomass produced (final and initial), initial pH, final pH, and initial theoretical alkalinity values of the experiments

Sample culture pH	Sample substrate^b^	Biomass (OD^c^)	Initial pH	Final pH	Initial alkalinity (mmol/liter)	Total acids produced^a^ (mmol/liter)
6.0	Glu	0.34 ± 0.03	5.99 ± 0.04	4.50 ± 0.22	3.54 ± 0.28	16.16 ± 2.13
	Fru	0.27 ± 0.03	5.95 ± 0.31	4.27 ± 1.19	3.30 ± 0.52	17.71 ± 0.69
	Cello	0.29 ± 0.03	5.95 ± 0.05	4.33 ± 0.03	3.23 ± 0.35	18.13 ± 0.61
6.5	Glu	0.35 ± 0.05	6.45 ± 0.13	6.13 ± 0.18	10.66 ± 2.99	12.54 ± 1.39
	Fru	0.39 ± 0.03	6.53 ± 0.04	6.39 ± 0.09	12.37 ± 1.04	13.83 ± 0.70
	Cello	0.49 ± 0.04	6.51 ± 0.02	4.57 ± 0.12	11.88 ± 0.64	16.19 ± 2.17
6.9	Glu	0.32 ± 0.02	6.86 ± 0.07	6.77 ± 0.01	26.82 ± 4.23	13.71 ± 1.20
	Fru	0.40 ± 0.02	6.92 ± 0.02	6.75 ± 0.06	30.08 ± 1.45	14.05 ± 0.51
	Cello	0.55 ± 0.01	6.94 ± 0.03	6.45 ± 0.05	31.75 ± 1.96	23.07 ± 0.23

a Measured after 72 h.

b Glu, Fru, and Cello indicate that the initial substrate was glucose, fructose, or cellobiose, respectively.

c OD, optical density.

Among the pH 6.5 cultures, Cello6.5 had twice as many electron equivalents; hence, those culture produced a higher concentration of acids, and they did not benefit from buffering as much as glucose and fructose cultures. The drop in the pH of the Cello6.5 cultures caused their community structure to resemble pH 6.0 cultures. pH 6.9 cultures provided sufficient buffering, and all three cultures developed similarly. The experimental design also probed the impact of alternative organic substrates (glucose or fructose) on community structure. Substrate type yielded no clustering pattern.

Figure 1A also shows the relative distributions of order-level phylotypes on the principal coordinates that clustered communities based on alkalinity and buffering. *Bacteroidales*, the most abundant order in the inoculum, was reduced in all cultures, while *Clostridiales*, *Lactobacillales*, and *Enterobacteriales* increased in all cultures. The main factors that separated the pH 6.0 cultures from the others were the greater abundance of *Lactobacillales* (>65%) and lower abundances of *Enterobacteriales* and *Bacteroidales*.

Besides beta diversity (the UniFrac metric), we calculated within-community diversity (alpha diversity) based on pH and substrate. Figures 1B and C portray the abundance-based coverage estimator (ACE) (33) and phylogenetic distance (PD) whole-tree (34) indices for richness and diversity, respectively. Compared to beta diversity, we observed a stronger substrate response on alpha diversity, and pH and substrate type had a combined effect on the alpha diversity indices. For glucose cultures, we did not observe a difference in diversity based on pH (for both ACE and PD whole tree). For fructose and cellobiose cultures, the ACE index showed that lower starting pH and alkalinity led to lower microbial richness. This trend was accentuated for cellobiose, a disaccharide composed of two glucose molecules, except when buffering was strongest at pH 6.9. The PD whole-tree index (Fig. 1C), a phylogeny-based diversity index, showed similar patterns as ACE. Thus, diversity (PD whole tree) was consistently higher at pH 6.9 than pH 6.0 and pH 6.5 for fructose and cellobiose cultures (Mann-Whitney U test; *P* = 0.02 and *P* = 0.04, respectively).

In summary, pH and substrate had a combined effect on within-community diversity. Sugars that likely reach the human colon (21, 30), like fructose and cellobiose, are important for maintaining microbial diversity in the human gut, as long as the pH is not substantially decreased.

Our results show that microbiota exposed to *in vitro*-relevant conditions responded to pH drops caused by limitations in the ambient buffering capacity, indicating the importance of alkalinity in stabilizing the human gut microbiota. A drop in gut pH due to increased microbial activity can lead to acidosis, a condition in which lactic acid accumulates in the bloodstream faster than it can be removed (35). The drop in pH, especially in pH 6.0 cultures, resulted in more lactic-acid producing bacteria (*Lactobacillales*) (36). Lactic-acid-producing bacteria exert beneficial effects on host health, such as promoting cholesterol absorption (37) and reducing diarrhea (38). Clinical studies have shown that mice treated with acidified water were less likely to develop diabetes than mice administered neutral-pH water, and the animals from these groups had differences in their gut microbiome structures (39).

### Acetate, lactate, and propionate accumulations depend on pH.

**FIG 2:**
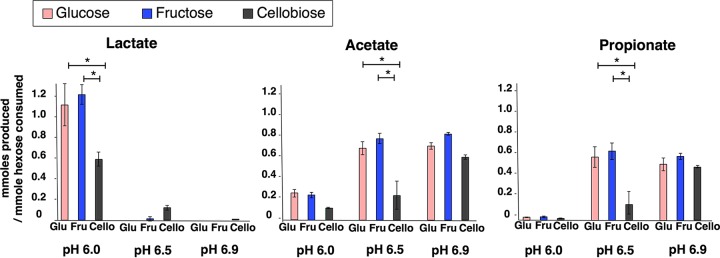
Major fermentation end products—lactate, acetate, and propionate—in mixed cultures fed glucose, fructose, or cellobiose at initial pH values of 6.0, 6.5, or 6.9. The millimoles of each acid produced was normalized per millimole of hexose consumed. Error bars represent the standard deviations of triplicates for each condition. *, Mann-Whitney U-test *P* value of <0.05.

Figure 2 shows the major short-chain fatty acids (SCFAs) in millimoles produced per millimole hexose consumed. Acetate and propionate were present in every culture, and they were the dominant end products of the pH 6.5 and pH 6.9 cultures. Acetate and propionate concentrations were 2- to 5-fold higher in pH 6.5 cultures and 7- to 27-fold higher in pH 6.9 cultures than in pH 6.0 cultures (*P* < 0.05). Lactate was the main end product of the pH 6.0 cultures but was undetectable or very low in all the pH 6.5 and 6.9 cultures. Lactate, which can be fermented to acetate and propionate (40), probably accumulated in the pH 6.0 cultures due to the acid stress on lactate-utilizing bacteria (41). Cellobiose cultures at pH 6.0 and 6.5 produced smaller amounts of fatty acids per hexose consumed (Fig. 2), due to incomplete fermentation (Table 2).

**TABLE 2:** Electron balances based on each metabolite’s electron equivalence^b^

Substrate, product, or parameter	Mean ± SD for culture with pH on substrate^c^								
	pH 6.0			pH 6.5			pH 6.9		
	Glu	Fru	Cello	Glu	Fru	Cello	Glu	Fru	Cello
Substrate	13.29 ± 0.9	13.59 ± 0.7	26.18 ± 3.5	13.10 ± 1.7	11.94 ± 0.9	24.31 ± 1.3	12.21 ± 0.5	11.83 ± 0.4	19.95 ± 1.1
Products									
Lactate	7.37 ± 1.3	8.24 ± 0.7	7.46 ± 0.9	0.00 ± 0.0	0.08 ± 0.1	1.61 ± 0.3	0.00 ± 0.0	0.00 ± 0.0	0.01 ± 0.0
Acetate	1.14 ± 0.2	1.10 ± 0.1	0.98 ± 0.1	2.80 ± 0.3	2.94 ± 0.2	2.14 ± 1.2	3.09 ± 0.2	3.27 ± 0.1	5.08 ± 0.2
Propionate	0.24 ± 0.0	0.28 ± 0.1	0.29 ± 0.1	4.32 ± 0.7	4.39 ± 0.5	2.40 ± 3.6	4.10 ± 0.5	4.26 ± 0.1	7.60 ± 0.2
Butyrate	0.48 ± 0.0	0.50 ± 0.1	0.43 ± 0.0	0.53 ± 0.1	0.60 ± 0.0	0.52 ± 0.1	0.61 ± 0.0	0.61 ± 0.0	0.75 ± 0.0
Formate	0.02 ± 0.0	0.10 ± 0.0	0.18 ± 0.0	0.03 ± 0.1	0.01 ± 0.0	0.10 ± 0.1	0.06 ± 0.0	0.01 ± 0.0	0.00 ± 0.0
Isobutyrate	0.03 ± 0.0	0.05 ± 0.0	0.03 ± 0.0	0.61 ± 0.9	0.18 ± 0.2	0.98 ± 0.4	0.10 ± 0.0	0.08 ± 0.0	0.12 ± 0.0
Valerate	0.08 ± 0.0	0.16 ± 0.0	0.22 ± 0.1	0.10 ± 0.0	0.18 ± 0.0	0.16 ± 0.1	0.20 ± 0.1	0.20 ± 0.0	0.16 ± 0.1
Isovalerate	0.04 ± 0.0	0.09 ± 0.0	0.25 ± 0.2	0.00 ± 0.0	0.06 ± 0.0	0.43 ± 0.3	0.00 ± 0.0	0.12 ± 0.1	0.09 ± 0.0
Citrate	0.40 ± 0.3	0.64 ± 0.4	1.42 ± 0.5	0.00 ± 0.0	0.02 ± 0.0	2.97 ± 1.9	0.00 ± 0.0	0.00 ± 0.0	0.01 ± 0.0
Hydrogen	0.08 ± 0.0	0.15 ± 0.0	0.02 ± 0.0	0.29 ± 0.0	0.15 ± 0.0	0.28 ± 0.4	0.13 ± 0.0	0.04 ± 0.0	0.04 ± 0.0
Ethanol	0.21 ± 0.0	0.21 ± 0.0	0.29 ± 0.0	0.68 ± 0.2	0.62 ± 0.0	0.67 ± 0.2	0.77 ± 0.2	0.69 ± 0.1	1.34 ± 0.1
Biomass	0.89 ± 0.1	0.76 ± 0.2	1.15 ± 0.3	1.22 ± 0.3	1.09 ± 0.2	1.86 ± 0.6	0.74 ± 0.3	1.07 ± 0.1	1.55 ± 0.7
RS^a^	0.00 ± 0.0	0.00 ± 0.0	13.21 ± 3.7	0.00 ± 0.0	0.00 ± 0.0	5.36 ± 4.6	0.00 ± 0.0	0.00 ± 0.0	0.00 ± 0.0
Total	10.76 ± 1.1	12.06 ± 0.9	25.63 ± 3.4	9.91 ± 0.9	9.69 ± 0.5	18.82 ± 1.0	8.65 ± 0.6	9.67 ± 0.43	15.42 ± 0.6
% recovery	80.90 ± 4.42	88.78 ± 5.2	97.96 ± 0.7	75.85 ± 3.2	81.22 ± 1.6	77.41 ± 0.7	73.12 ± 6.1	81.69 ± 3.07	77.38 ± 4.2

a RS, remaining substrate at end of experiment.

b Electrons for the biomass were determined based on the measured chemical oxygen demand. % recovery was calculated based on how much of the initial electron equivalents could be tracked by measurements at the end of the experiment.

c Glu, Fru, and Cello indicate that the initial substrate was glucose, fructose, or cellobiose, respectively. All concentrations and electron equivalents are those measured at the end of the experiment.

Minor fermentation products were butyrate, ethanol, and citrate (Table 2). Butyrate accumulation was minimal at all pH values. Similarly to lactate production, butyrate production is favored under acidic conditions, such as pH ≤5.5 (1), but butyrate producers require longer acclimation and incubation periods (42). Thus, low butyrate generation might be explained by the short experimental time of 72 h. Citrate, an intermediate in propionate and acetate fermentation (43), was detectable only in pH 6.0 cultures, as well as in the Cello6.5 cultures. Our results are consistent with those of Ramos et al., who showed that pH regulates citrate fermentation to acetate, the rate of which increases at higher pH (6.2 in comparison to pH 5) (44). Citrate accumulation in these cultures indicated incomplete acetate and propionate fermentation, and the presence of citrate and lactate explained low acetate and propionate concentrations in the pH 6.0 and Cello6.5 cultures. We did not observe this trend in the Glu6.5, Fru6.5, and pH 6.9 cultures, because the initial substrate was completely depleted, indicating that fermentation intermediates were converted into final fermentation products.

The electron mass balances in Table 2 show that 73 to 98% of the electrons from the initial substrates were captured by our SCFAs and biomass measurements. In our experiments, we used 10 mM organic substrate (glucose, fructose, and cellobiose) to achieve complete consumption for the majority of the cultures. This concentration is biologically relevant since 10 mM substrate yielded 7 to 16 mM SCFAs, which falls within the range observed in the distal colon (45). The unaccounted-for electron equivalents were likely present in amino acids and other metabolic products that were not measured. Electron balances verified that most electrons accumulated in intermediate molecules such as lactate and citrate when HCO_3_^−^ alkalinity was limiting, and electrons accumulated mainly in acetate and propionate when buffering with HCO_3_^−^ was sufficient.

Fermentation stoichiometry directly relates to fermentation reactions that occur in different parts of the human colon. For instance, the ascending and transverse colons with relatively lower pH (pH 5.4 to 6.2) (7) might experience greater lactate and less propionate and acetate accumulation than descending and rectosigmoid colons (pH 6.6 to 6.9) (7).

### Interactions between lactate-producing and -consuming microbial communities are driven by pH.

**FIG 3:**
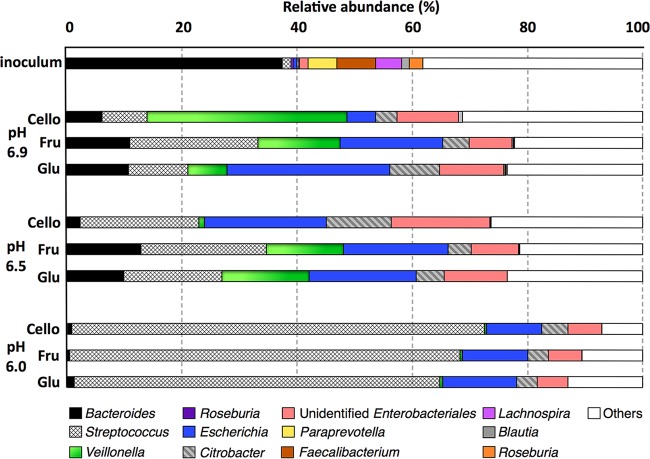
Relative abundance of phylotypes at the genus level in inoculum and fermentation cultures with initial pH values of 6.0, 6.5, or 6.9 and with glucose, fructose, or cellobiose as the initial substrate.

**FIG 4:**
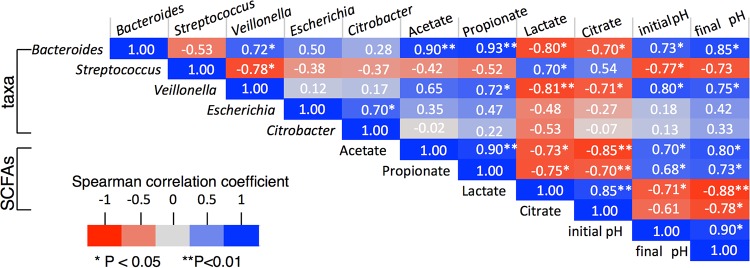
Nonparametric correlation coefficients (Spearman’s rank) between combinations of taxa, initial pH, and fermentation end products.

Figure 3 shows that the inoculum consisted of diverse butyrate-producing species, including *Faecalibacterium* (6.8%), *Roseburia* (2.4%), and *Lachnospira* (4.7%). However, these butyrate producers were almost eradicated by batch culturing, and the low abundances of these phylotypes in the resulting cultures (<1%) can explain the observed low butyrate yields. In contrast, genus-level phylotypes, including *Citrobacter*, *Escherichia*, *Streptococcus*, and *Veillonella*, were present in low abundances in the inoculum but became the majority of the phylotypes in the cultures. As shown in Fig. 4, *Veillonella* and *Bacteroides* were positively correlated, whereas *Streptococcus* was negatively correlated, with initial and final pHs.

Substantial differences in lactate, propionate, and acetate production during fermentations at various pHs prompted us to investigate the role of pH in lactate-producing and -utilizing communities. These genus-level phylotypes were mainly from *Lactobacillales*, *Enterobacteriales*, *Clostridiales*, and *Bacteroidales* orders, which drove the cultures with limited buffering away from sufficient buffering (Fig. 1). We examined phylotypes similar to dominant genera of lactate-producing *Streptococcus* (46) and *Citrobacter* (47), lactate-utilizing *Veillonella* (48), and lactate-producing and lactate-utilizing *Bacteroides* (49) and *Escherichia* (50, 51). The initial pH and the buffering capacity (affecting the final pH) had substantial impact on the relative abundance of these phylotypes. Only when buffering was sufficient (at pH 6.5 and 6.9) did we observe that the substrate type had secondary effects on phylotype abundance (similar to alpha diversity indices).

Lactate-producing *Streptococcus* phylotypes comprised only 1.5% of the inoculum, and they increased to 61 to 73%, 17 to 20%, and 7 to 22% in the pH 6.0, 6.5 and 6.9 cultures, respectively (Fig. 3). Abundances of *Citrobacter*, another lactate producer, did not mimic the trend of *Streptococcus*, as it thrived in Cello6.5 cultures. Figure 4 shows nonparametric correlation coefficients for the associations between fermentation end products, pH, and major microbial phylotypes. The abundance of *Streptococcus* phylotypes strongly and positively correlated with lactate concentration (Spearman’s rank = 0.70, *P* < 0.05), and the dominance of *Streptococcus* phylotypes at pH 6.0 can explain lactate accumulation in these cultures. *Citrobacter* did not significantly correlate with the abundance of lactate or citrate (Spearman’s rank correlation coefficients for lactate and citrate = −0.53 and −0.07, respectively; *P* > 0.05), possibly because citrate is a fermentation intermediate in *Citrobacter*, which gains energy from fermenting glucose to citrate and lactate and subsequently fermenting citrate and lactate to acetate (52).

*Veillonella*, a reported lactate utilizer (48), made up less than 1% of the inoculum and pH 6.0 cultures; its low abundance in the pH 6.0 cultures could be due to poor acid tolerance (48). *Veillonella* phylotypes varied from 1 to 35% in the pH 6.5 and 6.9 cultures, with the highest prevalence in the Cello6.9 (35%) and the lowest in the Cello6.5 (~1%) culture. Unlike *Bacteroides*, *Veillonella* lacks the ability to ferment sugars, but it ferments organic acids, such as lactate and pyruvate, to propionate and acetate (48). The abundance of the *Veillonella* phylotype had a negative correlation with lactate accumulation (Spearman’s rank correlation coefficient = −0.81, *P* < 0.01) and a positive correlation with propionate accumulation (Spearman’s rank correlation coefficient = 0.72, *P* < 0.05).

*Bacteroides* phylotypes were 37% of the inoculum, but they declined to less than 2% in the pH 6.0 cultures and 2 to 12% of the pH 6.5 and 6.9 cultures. This observation is consistent with *Bacteroides* having weak acid tolerance (1). The abundance of *Bacteroides* phylotypes correlated negatively with lactate concentration (Spearman’s rank correlation coefficient = −0.80, *P* < 0.05) but positively with acetate and propionate concentrations (Spearman’s rank correlation coefficient = 0.93, *P* < 0.01). Along the same lines, high lactate accumulation and low accumulations of acetate and propionate in the Cello6.5 culture can be explained by the low relative abundances of the lactate consumer and propionate producers *Veillonella* and *Bacteroides*. The lower abundances of *Bacteroides* and *Veillonella* phylotypes in pH 6.0 cultures happened in concert with lactate accumulation.

*Escherichia* was relatively abundant in all cultures (5 to 28%), especially for pH 6.5 and 6.9. The high abundance of *Escherichia* at pH 6.5 and 6.9 suggests that it replaced *Streptococcus* in the lactate production niche. *Escherichia* is able to either produce or consume lactate, and this metabolic versatility may explain why it was a key member of all culture communities. Lactate-utilizing and acetate- and propionate-producing bacteria such as *Bacteroides*, *Escherichia*, and *Veillonella* and butyrate-producing microorganisms such as *Faecalibacterium* and *Roseburia* are essential for removing accumulated lactate in the colon (53). An absence of lactate consumers in the colon can lead to reduced pH of the colon via d-lactic acidogenesis, and the consequence of lower pH is a deterioration of the host’s health (54).

These microbial composition results have implications for spatial distribution of microbiota in the gastrointestinal tract that consume simple carbohydrates and are related to health conditions such as bariatric surgery, inflammatory bowel disease, and colorectal cancer. Since we studied only carbohydrate fermenters, our results are probably more applicable to the ascending and transverse colons, where the greatest carbohydrate fermentation occurs (55). We speculate that acid-tolerant species, such as some *Streptococcus* strains and enteric bacteria, flourish in the ascending and transverse colons, where the pH is slightly lower than in the descending and rectosigmoid colons (7). *Bacteroides* species may dominate in the descending and rectosigmoid colons, where the pH is slightly higher (7).

The microbiota compositions at pH 6.5 and 6.9 were similar to the reported colonic microbiota of post-Roux-en-Y gastric bypass (RYGB) surgery (56) and colorectal cancer patients (57). RYGB surgery enriched *Gammaproteobacteria* phylotypes (56), more specifically *Escherichia* (58, 59) and *Citrobacter* (60), as well as phylotypes most closely related to *Veillonella*, a lactate-consuming propionate-producing member of the *Firmicutes* phylum (60). Because this weight loss surgery reduces gastric acid secretions, it might select for less-acid-sensitive microorganisms (61) and increase fecal propionate concentration (62). In colorectal cancer patients, enrichment of many genera in the colon, such as *Streptococcus*, *Enterococcus*, *Escherichia*, *Klebsiella*, and *Peptostreptococcus*, has been observed (63), and these correspond to genus-level phylotypes detected here at relatively higher pH. In irritable bowel disease patients, higher colonic pH was observed (64), and microorganisms from *Bacteroides* and *Veillonella* occurred in greater abundance in these subjects than in healthy individuals (65).

A limitation of this study is that we utilized batch bottles and enriched for microbial species with a single carbon source, which does not represent the complexity of the human gut. Nevertheless, findings of our study are useful for interpreting instances in which the pH of the intestine drops due to limiting buffering capacity.

We studied how pH, alkalinity, and carbohydrate substrate affect the microbial community structure and function of a mixed-culture inoculum taken from the stool of a healthy human. Low pH, caused by limited bicarbonate alkalinity, had by far the strongest impact on community structure and metabolism. Impacts of substrate type on microbial community structure were secondary and evident only when alkalinity was not sufficient. Thus, a transient shift in pH from 6 to ~4 led to a less-diverse microbial community that formed less acetate and propionate but more lactate. As a consequence of limited buffering, a drop in the pH disrupted the growth of some community members, hence the restrained microbial and metabolic interactions between lactate-producing and lactate-utilizing communities.

## MATERIALS AND METHODS

### Experimental design.

We obtained Institutional Review Board (IRB) approval from Arizona State University (IRB number 1203007553). A fecal specimen was collected from a healthy female subject and transported to the laboratory on ice packs. After homogenizing 1 g of the specimen in 50-ml sterile anaerobic 1× phosphate-buffered saline at pH 7.2, we produced the fecal slurry used in the experiments. The inoculum was diluted to a final concentration of 0.04 g/liter solids, and all inoculations were carried out in an anaerobic glove box.

The culturing medium was an anaerobic fermentation medium (66) that contained 30 mM sodium bicarbonate (NaHCO_3_), 2% cysteine-sulfide solution, and 10 mM one fermentable substrate (glucose, fructose, or cellobiose). Glucose and fructose are monosaccharides that have the same electron equivalence (24 electrons per mole), although they have different chemical properties and metabolism by bacteria (67). By comparing glucose and fructose fermentations, we were able to identify microbial diversity and metabolic pathways that are dependent on monosaccharide variety and availability rather than the number of electrons available for bacterial metabolism. Because the cultures had the same millimoles of substrate in the batch bottles, cellobiose cultures received twice the amount of the electrons that fructose or glucose cultures received, since cellobiose is a disaccharide. By comparing cellobiose to glucose fermentation, we were able to understand the effects of electron availability on microbial metabolism and community structure at different pH values.

After preparing the medium anaerobically under a stream of 20/80% CO_2_-N_2_ gas, we distributed 50 ml of medium into triplicate 125-ml serum bottles and then adjusted the pH to 6.0, 6.5, or 6.9 with 10% hydrochloric acid. Before inoculation, we flushed the headspace with 20/80% CO_2_-N_2_ gas and equilibrated the contents to atmospheric pressure (1 atm). We labeled the cultures based on their initial pH and substrate: Glu6.0, Glu6.5, Glu6.9, Fru6.0, Fru6.5, Fru6.9, Cello6.0, Cello6.5, and Cello6.9.

All inoculated bottles were incubated at 37°C in a shaking incubator (New Brunswick Scientific, Enfield, CT) at 150 rpm. The duration of the experiment was 72 h: the first 24 to 48 h to reach stationary phase and establish biomass and another 24 h to ferment substrate. We sampled the liquid and gas phases at 0 h and 72 h. All conditions were reproduced in triplicate, and the means and standard deviations of the triplicates are reported.

### Growth and fermentation end product measurements.

We documented growth by measuring optical density at 600 nm (Varian Cary 50 Bio UV) and pH using a pH meter (Thermo Scientific Orion). We sampled the liquid phase at the time of inoculation and at the end of 72 h using sterile syringes equipped with sterile 20-gauge needles and filtered the supernatant through 0.2-µm polyvinylidene difluoride (PVDF) membranes (Acrodisc; LC 13-mm syringe filter).

We analyzed substrates and metabolites using a high-pressure liquid chromatograph (HPLC) (LC-20AT; Shimadzu) equipped with a carbohydrate column (Aminex HPX-87H column; Bio-Rad) as previously described (66). Short-chain fatty acids (acetate, formate, butyrate, isobutyrate, isovalerate, valerate, propionate, and lactate) and alcohols (ethanol and methanol) were analyzed using 5 mM H_2_SO_4_ as the eluent, an 0.6-ml/min flow rate, a column temperature of 50°C, and a 50-min run time. The carbohydrates (glucose, fructose, and cellobiose) were analyzed using 18-ohm water as eluent, a 0.6-ml/min flow rate, a column temperature of 30°C, and 30 min of run time. The SCFAs and alcohols were detected with a photodiode array (PDA) detector (Shimadzu), and the sugars and alcohols were detected with a refractive index detector (RID; 10A; Shimadzu). We normalized the millimoles of SCFAs produced to millimoles of hexose consumed.

In order to perform electron-equivalent mass balances, we measured the total chemical oxygen demand (COD) of the samples before filtering and soluble COD after 0.2-µm filtration using a Hach COD analysis kit (Hach Co., Loveland, CO). We calculated the electron equivalents of sugars, fermentation end products, and biomass using the stoichiometric equations as specified in the work of Rittmann et al. (68). We also calculated theoretical alkalinity based on initial pH, partial pressure of CO_2_, and pK_a_ of the HCO_3_^−^ using the equation specified in the work of Rittmann et al. (68). The calculated pK_a_ of HCO_3_^−^ was 6.16 when the ionic strength of the medium was 0.03.

### DNA extraction and sequencing.

We extracted DNA from the inoculum and the resulting mixed fermentative consortia using a QIAamp Mini stool kit (Qiagen, CA) and followed the manufacturer’s recommendation for pathogens with minimal modification. Briefly, we incubated the lysis solution and bacterial mix at 95°C to enhance the lysis of Gram-positive bacteria. We verified the quantity and quality of DNA samples using a NanoDrop instrument and by measuring the absorption at 260 and 280 nm. We stored the extracts at −80°C until sequencing.

We amplified genomic DNA with a barcoded primer set targeting the V2-V3 regions of 16S rRNA genes (69). Sequencing libraries were prepared according to the work of Claesson et al. (70), and purified PCR products were sent to the DNASU Genomics Core Facility at the Virginia G. Piper Center for Personalized Diagnostics in the Biodesign Institute at Arizona State University (Tempe, AZ), which provided pair-end reads (2 × 100 bp) using the HiSeq2000 platform (Illumina Inc., San Diego, CA). We received fastq files and deposited the sequences into the Sequence Read Archive.

### Sequence analysis.

We analyzed data using the QIIME 1.8 suite (71). We filtered the sequences using default values and by setting the minimum quality score to 21 and minimum length to 192. We clustered sequences into operational taxonomic units (OTUs) at the 97% level of sequence similarity using Uclust (72), picked the most abundant sequence as representative of each cluster, and then assigned taxonomy to the sequences using the RDP algorithm at a 50% threshold (73) and the Greengenes Database 2013 release (74). We aligned representative sequences using PyNAST (75) and identified chimeric sequences with ChimeraSlayer (76). We calculated within-sample (alpha) diversity indices: phylogenetic distance whole tree (34) for diversity and ACE (33) for richness. The weighted UniFrac metric (32) was used to calculate intersample diversity (beta diversity).

### Statistics.

Since our data size is small (*n* = 3 per group), nonparametric tests were more suitable for our data sets. We used the Mann-Whitney U test for significance and accepted *P* values less than 0.05 as significant. To find relationships between pH, microbial phylotypes, and metabolic end products, we performed the Spearman correlation test and accepted correlation coefficients with *P* values of <0.05 as significant associations. All the statistical procedures were carried out with Statistical Package for Social Sciences version 22. Using QIIME (71), we performed ANOSIM analysis (77), a similarity test on distance matrices, with 9,999 permutations.

### Accession number(s).

We deposited the sequences in the Sequence Read Archive under accession numbers SAMN03120391 to -400.
